# Drivers of the likelihood of evolutionary rescue in response to novel infectious diseases

**DOI:** 10.1073/pnas.2614169123

**Published:** 2026-10-15

**Authors:** Laurinne J. Balstad, Marissa L. Baskett

**Affiliations:** ^a^ Department of Environmental Science and Policy, University of California, Davis, CA 95616; ^b^ Center for Population Biology, University of California, Davis, CA 95616

**Keywords:** evolutionary rescue, mathematical model, adaptation, host-pathogen system, disease

## Abstract

The introduction of novel infectious diseases has caused significant declines in many species, and in some cases, local extinction. Simultaneously, hosts might adapt to these novel pathogens, allowing population recovery, an example of evolutionary rescue (ER). Novel infectious diseases differ from sudden, abiotic stressors in that disease only impacts a subset of individuals within the population and individuals might be affected for a finite period. Whether ER occurs in an infectious disease context, and how likely it is relative to abiotic stress, might depend on both host and pathogen characteristics such as the scale of transmission (e.g., local density-dependent transmission only or additional infection through an external reservoir), the degree of fatality or immunity, and the adaptive pathway in terms of whether adaptive alleles reduce mortality, reduce likelihood of infection, or enhance recovery. Here, we use a stochastic mathematical model of host-pathogen dynamics with evolution of a novel allele conferring disease robustness to quantify how population trajectories, including the probability of and time to ER, vary across adaptive pathways and disease characteristics. We find that ER is more likely to occur with dynamical disease transmission compared to a lasting stressor that affects the entire population, indicating that protecting a subset of the population from stress facilitates ER. Among cases with disease transmission, ER is generally more likely to occur with the adaptation of mortality-blocking alleles and sources of transmission that include an external reservoir.

## Significance

When novel environments cause population decline, evolution can reverse this decline (“evolutionary rescue;” ER). Examples of ER include hosts adapting to novel pathogens that could otherwise lead to extinction. We develop predictive theory exploring the ecological and evolutionary conditions under which adaptation to a novel infectious disease might enable ER and conditions under which extinction risk is higher. Our work demonstrates that protecting some individuals from stress can increase ER despite potentially slowing selection, building on past work showing that reduced stress, for all individuals, increases ER. Managers can leverage the range of simulations here to identify strategies, including pathogen extirpation and reduction of additional anthropogenic stressors, to increase the likelihood of population recovery following novel infectious disease outbreaks.

Emerging, novel infectious diseases have caused significant declines of host populations across systems of ecological, social, and economic importance (1, 2). For example, Tasmanian devils (*Sarcophilus harrisii*) have experienced local declines of up to 90% largely due to Devil Facial Tumor disease, causing subsequent turnover and biodiversity loss in the broader ecological community (3). Various amphibians (4, 5), little brown bats [*Myotis lucifugus*; (6, 7)] and sea stars [including *Pisaster ochraceus*; (8, 9)] have similarly declined rapidly due to novel infectious disease outbreaks. Outside conservation contexts, diseases in livestock, aquaculture, and agriculture threaten food security and economic productivity [e.g., African swine fever virus; (10)]. Through exposure, hosts might evolve robustness to emerging diseases, allowing them to persist despite possible extinction risk. This process is known as evolutionary rescue [ER; (11, 12)]. Examples of disease-induced ER have been documented more frequently for wild animal populations compared to ER following other sources of stress (13). Such cases include the evolution of immunity to Devil Facial Tumor disease in Tasmanian devils (14–17) and the evolution of tolerance to white nose syndrome in bats (6, 7). Various amphibians (18–20), abalone [*Haliotis cracherodii*; (21, 22)], prairie dogs [*Cynomys ludovicianus*; (23, 24)], sticklebacks [*Gasterosteus aculeatus*; (25)], and rabbits [*Oryctolagus cuniculus*; (26)] similarly show evidence of infectious disease-induced adaptations. However, ER in response to infectious disease is not a given: In many populations, there is no clear variation in disease response and disease-induced population declines have resulted in local extinction [e.g., Bighorn sheep (*Ovis canadensis*), Spanish ibex (*Capra pyrenaica hispanica*), and black-footed ferret (*Mustela nigripes*); (2)].

**Fig. 1.**
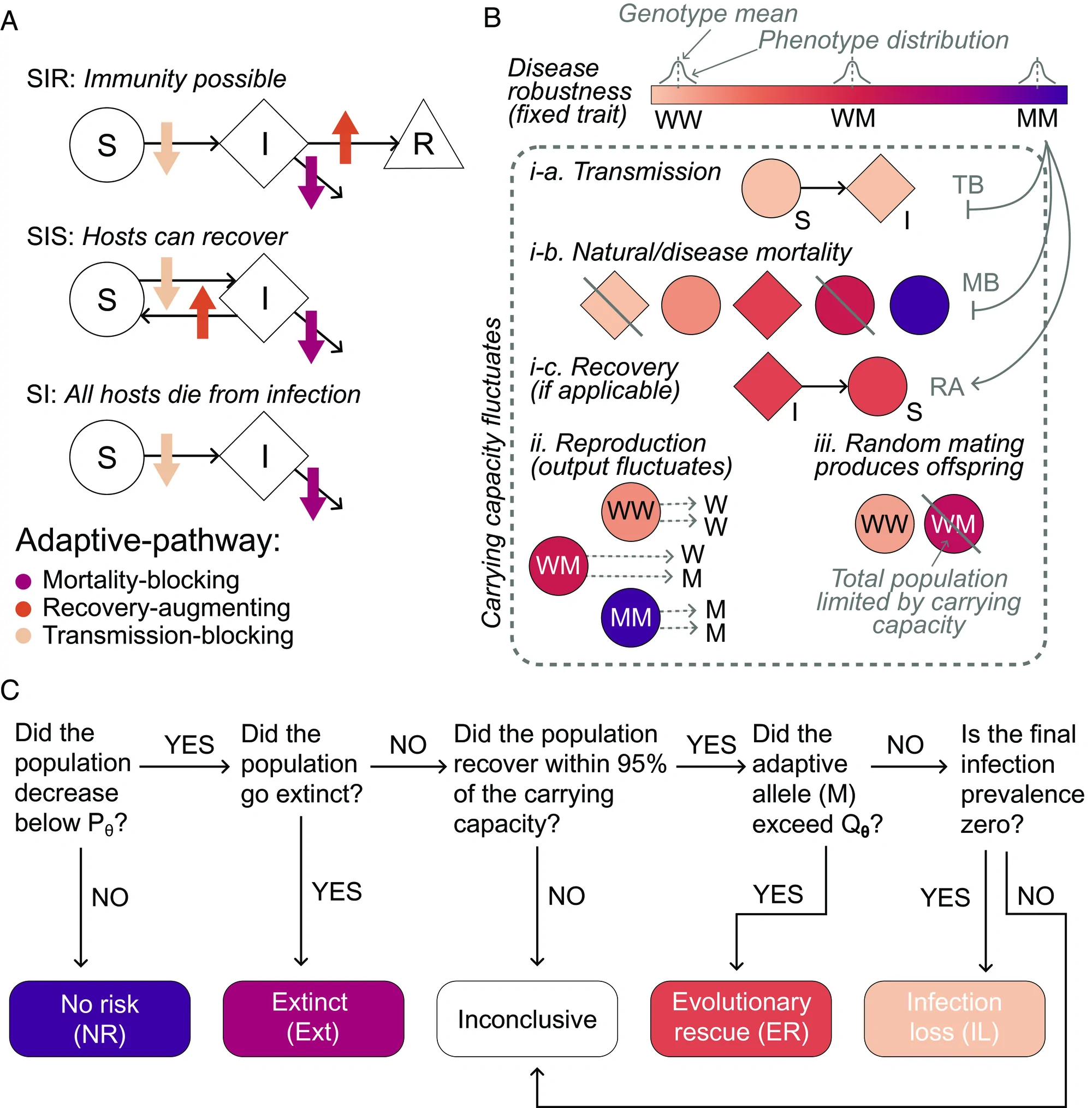
(*A*) Schematic demonstrating compartment structures and how different adaptive pathways shape the likelihood of transitioning between susceptible (S), infected (I), and recovered (R) class, when applicable. Dark pink arrows illustrate mortality-blocking alleles that reduce the likelihood an infected individual dies, dark orange arrows illustrate recovery-augmenting alleles that increase the likelihood an infected individual recovers, and light cream arrows illustrate transmission-blocking alleles that reduce the likelihood of disease transmission. (*B*) Adaptive alleles (M, dark pink to dark purple shapes) affect disease transmission rates, virulence rates, and recovery rates, compared to wild-type alleles (W, light cream shapes). Individual robustness against disease, as the proportional change in the mean transmission rate (decrease in rate), mortality rate (decrease in rate), and recovery rate (increase in rate), is fixed for the individual’s lifetime. Circles indicate an individual is susceptible to disease, while diamonds indicate the individual is infected. After the population reaches a quasi-equilibrium, we introduce disease. Here, we show ordered disease dynamics of transmission (Bi-a within dashed box), mortality (Bi-b within dashed box), and recovery (Bi-c within dashed box), but we test alternative orderings in *SI Appendix*, Fig. S5. Adaptive alleles can either decrease the likelihood of the transition occurring (shown by the capped arrows; TB = transmission-blocking, MB = mortality-blocking) or increase the likelihood of transition occurring (shown by the arrow; RA = recovery-augmenting). Next, surviving individuals reproduce, regardless of infection status (Bii within dashed box). Alleles are combined to create offspring, where the total number of offspring is limited by a time-varying carrying capacity (Biii within dashed box). (*C*) Flowchart shows how population-level outcomes are classified, based on the threshold of population decline ($P_{\theta }$), the threshold for adaptive allele peak ($Q_{\theta }$) and the final infection prevalence ($I_{t}$). We combine “Permanent” and “Temporary” ER, resulting in five possible population outcomes.

Unlike a sudden, abiotic stress, infectious disease, and hence disease-induced selection, only impacts a subset of individuals within the focal population at a given time and, in some cases, impacts individuals for a finite period of their life. On one hand, this could increase the overall population growth rate and thus the likelihood of ER and population persistence. On the other hand, fitness differences between nonadapted and adapted individuals [i.e., selection gradient; (27)] might be smaller in host-pathogen systems, making ER less likely. In the face of these competing processes, the relative likelihood of ER in response to disease might depend on an array of disease and host ecological characteristics that shape selection and population dynamics, such as the scale of disease transmission and host response potential (28, 29); Fig. 1*A*]. When disease transmission depends only on local individuals (“Density only” case), selection pressure could change across time and disease can be extirpated for a closed population. In contrast, if there are external reservoirs (“Density w/reservoir” case), disease can re-emerge in the absence of presently infected individuals. Similarly, transmission probability and selection pressure might change over time as individuals leave the infected class. These changes might depend on the impacts of infection on a host (e.g., if all infected hosts die from infection (SI compartment structure; using the disease dynamics representation of S for susceptible, I for infected, R for recovered), if hosts can recover and reenter the susceptible class (SIS compartment structure), or if hosts recover and acquire immunity after infection (SIR compartment structure), among others (SIRS, etc.). This results in different degrees of selection because both risk of infection (transmission) and degree of infection (disease-induced mortality), on average, vary across these different disease ecology cases (29).

Whether and how infectious disease induced ER differs from ER following a sudden, abiotic stress could further depend on two characteristics of the traits under selection (30–34). First, the degree of protection against disease (“robustness”) provided by an adaptive allele and the costs associated with an adaptive allele might vary across systems. Alleles that provide partial benefits or which incur a high cost could slow the speed of evolution, preventing ER. Second, the mechanism by which traits confer disease robustness [here “adaptive pathways,” *sensu* (35)] might affect the degree to which evolution affects future infection prevalence and selection. For example, alleles conferring disease resistance (transmission-blocking) reduce the risk of infection but do not necessarily reduce disease-induced mortality. In contrast, alleles conferring disease tolerance (mortality-blocking) reduce disease-induced mortality without reducing the likelihood of infection, allowing continued disease spread. Alleles that increase disease clearance rates (recovery-augmenting) fall between these two cases by indirectly reducing disease mortality and, when immunity is possible, reducing future risk of infection. In all cases, infection dynamics change with adaptation, which then affects the selection individuals experience for further disease robustness (28, 33, 36). This differs from selection by lasting, abiotic stressors that induce an evolutionary response that increases the fitness of adaptive allele carriers without impacting future stress and selection (11, 37). In addition, the adaptive pathway can also affect the time to population recovery, where past theory predicts tolerance-blocking alleles can increase the time to recovery relative to other adaptive pathways (34).

Past modeling work on disease-induced ER, primarily developed for case-specific contexts (6, 38–42), highlights that disease can lead to ER, extinction, or nonadaptive infection loss (IL; reviewed by ref. 36). As with ER in response to abiotic stressors, these models suggest ER occurs when transmission and mortality rates are high enough to cause population decline and selection without leading to a very high probability of extinction. When transmission and mortality rates are too strong, either the host goes extinct or the pathogen is extirpated (38, 39, 42). Such case-specific models are one component of a predictive theory for ER, as the biological realism of this theory enables a direct comparison of predictions to empirical patterns. Complementing this modeling approach, a more generic, system-agnostic approach with functional and parameter sensitivity analysis provides another aspect of predictive theory by elucidating the drivers of and host-pathogen systems under in which ER is more likely to occur. Past work aiming to create a general framework predominantly focuses on a subset of the disease ecology-adaptive pathway space, either considering the effect of alternative adaptive pathways for a fixed host response to infection (34) or the effect of multiple host responses considering only one adaptive pathway (42). The next step is then to understand the interaction between these two factors, using a probabilistic framework to quantify and predict the relative likelihood of host extinction, ER, and IL across various scenarios. Such an approach could provide a comprehensive and integrative understanding of the conditions under which disease-induced ER is more or less likely to occur and how selection pressures and adaptive pathways affect the progression of ER. In addition, these comparisons can elucidate how ER unfolds in infectious disease systems, for which there is a dynamic selection pressure with several possible adaptive pathways, compared to other abiotic stressors, for which selection pressure is constant and impacts all individuals simultaneously.

Here, we used an individual based model to quantify how different infectious disease and host characteristics affect the likelihood of ER in host-pathogen systems (Fig. 1 *A* and *B*). First, we tested how host response potential (mortality only, recovery, immunity), scale of transmission, and type of adaptive pathway affect the likelihood of extinction, ER, and IL. We compared these scenarios to a counterfactual in which all individuals are immediately infected, analogous to sudden and lasting abiotic stress (“Lasting disease shock” case). This helps tease apart how the nature of disease-induced selection, where a subset of the population is temporarily stressed, compares to a lasting and sudden abiotic stress in the context of ER. Second, we considered how the relative benefit and cost of the adaptive allele shape the likelihood of disease-induced extinction, ER, and IL. Third, given that large populations are more likely to experience both disease outbreaks and ER, we quantified the effect of population size on the likelihood of disease-induced ER. Finally, we conducted a global sensitivity analysis [GSA; (43)] to identify the model parameters that exerted the greatest influence on the probability of extinction, ER, and IL. An understanding of the characteristics and parameters that facilitate ER will enable researchers to predict which outcome a given population might experience.

## Methods

We model a host-pathogen system that includes infectious disease trans-mission, mortality (natural and disease-induced), disease recovery, and acquired immunity (when applicable, Fig. 1 *A* and *B*). We implement this model as an individual-based simulation with demographic stochasticity in order to capture the effect of such stochasticity on extinction risk and drift (fixation or loss of disease-resistant alleles) in small populations. Fixed characteristics of individual hosts (j) include genotype (G), which determines the rate of disease transmission, mortality, and recovery. Reproductive output, also influenced by genotype, and infection status (T∈ (susceptible/S, infected/I, recovered/R)) can vary each time step (mathematical details below and in *SI Appendix*). We denote each individual by its combined genotype and infection status, $j_{G,T}$. In addition, we incorporate environmental stochasticity by varying carrying capacity ($K_{t}∼N\left(K,\sigma_{K}^{2}\right)$, which affects the maximum number of offspring that survive.

We assume disease robustness is the result of a single, pleiotropic gene, which determines the probability of an individual becoming infected (scaled by density, when relevant), dying from disease, recovering from disease (if applicable) and, to account for the costs of robustness against disease, the average reproductive output of the individual. Wild-type alleles (W) correspond with nonadaptive disease rates, while mutant-type alleles (M) can be transmission-blocking (i.e., reduce the probability that an individual becomes infected), mortality-blocking (i.e., reduce the probability that an individual dies from disease), and/or recovery-augmenting (i.e., increase the probability that an infected individual recovers). The mutant-type allele, conferring robustness against disease, changes in frequency through selection, drift, and mutation during reproduction. An individual’s likelihood of experiencing a given event (transmission, death, or recovery) is converted to phenotype through a draw from a truncated-normal distribution of the genotype mean to account for imperfect inheritance and environmental variance affecting phenotypic expression.

We define the allele’s benefit as the percent change in event rate, relative to homozygous wild-type individuals (WW), experienced by homozygous mutant individuals (MM) [converted to probability for discrete time (44); Fig. 1*A*]. The allele’s benefit to heterozygote individuals (MW) was determined by the adaptive allele’s dominance. Note “benefit” results in a decreased event rate for transmission- and mortality-blocking alleles, and an increased rate for recovery-augmenting alleles. We define the allele’s cost as the percent reduction in fecundity, relative to homozygous wild-type individuals, for homozygous mutant individuals. For the counterfactual case (“Lasting disease shock”), the probability of infection for wild-type individuals is one, the probability of mortality is matched to disease-induced mortality and the probability of recovery for wild-type individuals is zero. In other words, infection is instant, affects all wild-type individuals and is lasting. Detailed methods, including equations describing the transmission, mortality, and recovery events, are in *SI Appendix*.

To track time within the simulation, we use disease generations to explore how the relative pace of disease and host life history affect model outcomes. Specifically, we consider d disease generations between host reproduction events. For example, if there are 150 disease generations and d=2 disease generations between host reproduction events, then there are 150 disease generations and 75 host reproduction events. During each disease generation, discrete infection, mortality, and recovery transition events are determined by a weighted coin flip, followed by reproduction, where the number of expected offspring is taken from a stochastic draw around the individual’s phenotype for reproductive output (Fig. 1*B*). At the end of each disease generation, we track the number of individuals ($P_{t}=\sum j_{G,T}$), the infection prevalence ($V_{t}=\sum j_{G,T=I}/P_{t}$), and the frequency of the M allele ($Q_{t}=\left(2*\sum j_{G=MM,T}+\sum j_{G=WM,T}\right)/\left(2*P_{t}\right)$). To identify the time to recovery via ER, we use the host generation for which the population first reaches 95% of the carrying capacity (K) after the population minimum.

We simulate each parameter combination 2,500 times and summarize the population-level outcomes to understand the likelihood of each outcome (i.e., extinction, ER, and IL; Fig. 1*C*). Each simulation begins with a disease-free period to allow for balance between mutation and selection. In addition to targeted simulations, we conduct a multivariate global sensitivity analysis [GSA; (43)] to identify the factors that most influence the relative likelihood of each outcome. A detailed description of simulation experiments and GSA methods are in *SI Appendix*.

We categorize the simulation outcomes based on 1) whether the population has decreased relative to the carrying capacity, 2) whether the population has gone extinct or, after decreasing to its minimum, has recovered to near the carrying capacity, 3) whether the M allele has increased in frequency at some point during the simulation and 4) whether the disease persists or has been extirpated (Fig. 1*C*). We classify trajectories as ER when a population 1) declines below the a priori selected threshold, $P_{\theta }=0.5$ K, for at least three time steps and stays below the threshold for 80% of the time steps between the first and last occurrence of declining below $P_{\theta }$, 2) recovers to at least 0.95 K after reaching the population minimum and 3) when the frequency of the M allele increases above some a priori selected threshold, $Q_{\theta }=0.45$. We subclassify ER as either “Persistent” or “Temporary” based on the final disease prevalence and the final M allele frequency; see below for details. We classify trajectories as IL when a population 1) declines below $P_{\theta }$ for at least three time steps and stays below the threshold for 80% of the time steps between the first and last occurrence of declining below $P_{\theta }$, 2) recovers to at least 0.95 K after reaching the population minimum, 3) when the frequency of the M allele remains below $Q_{\theta }$ and 4) when disease is extirpated ($I_{t=t_{f}}=0)$. In some simulations, populations do not experience extinction risk, either because the disease does not establish (due to stochasticity) or because the infection is not detrimental at the population scale (due to low transmission or virulence). These two types of trajectories are combined into a “no risk” (NR) category reflecting that the introduction of disease did not lead to population decline. In other simulations, populations experience long transience (i.e., do not recover or go extinct during the model simulation period, $t_{q}+t_{f}$). We classify these trajectories as “inconclusive.”

## Results

**Fig. 2.**
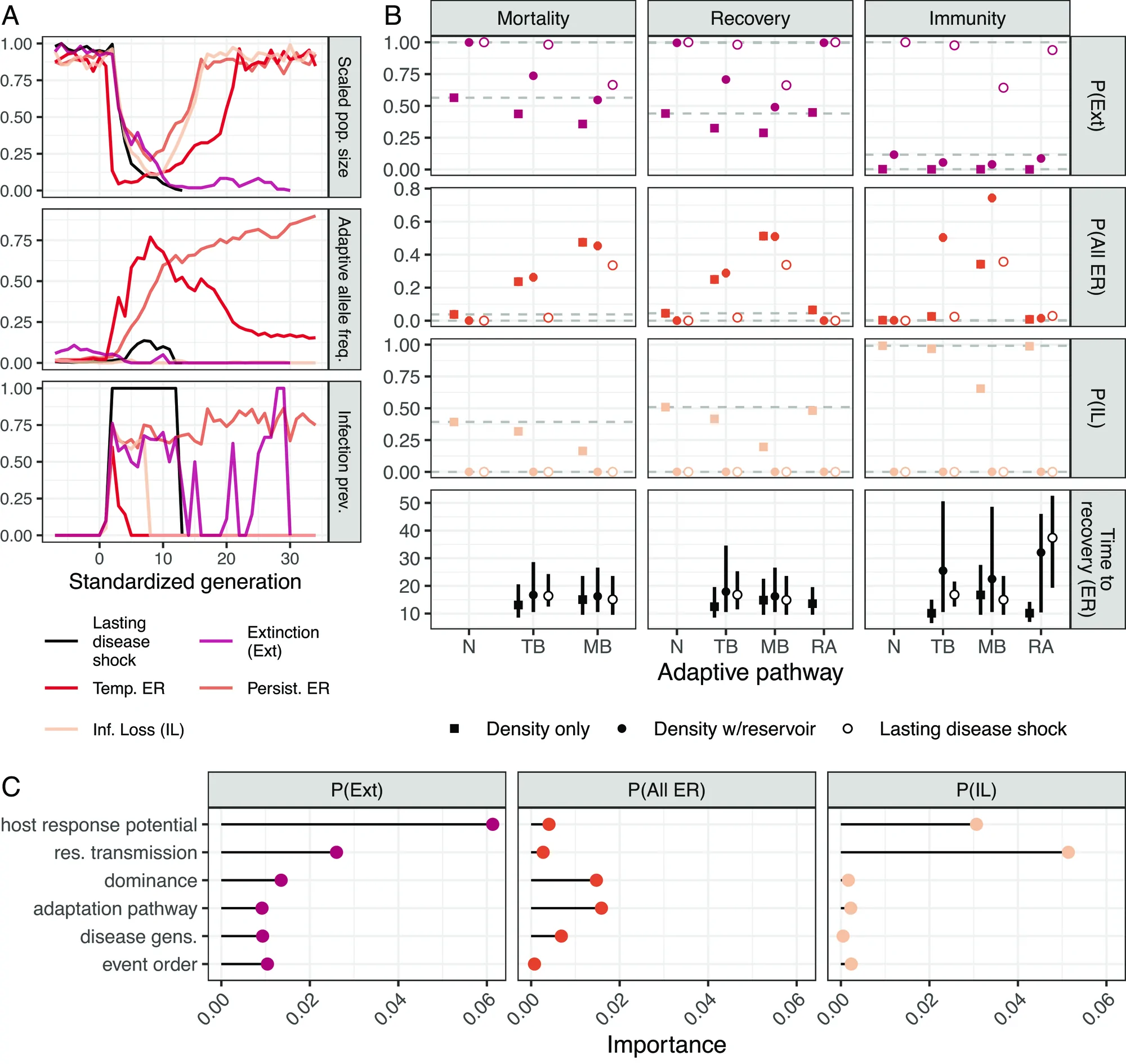
(*A*) Example population trajectories, by color, described by the population size, the adaptive allele frequency, and the infection prevalence. (*B*) Probability of population outcomes (point color) under different adaptive pathways: extinction (*Top* row, dark pink), ER (including both persistent and transient trajectories; second row, dark orange), IL (third row, light cream), and time to recovery via ER (*Bottom* row, black). The x-axis denotes types of adaptive pathways: “N” for no adaptation, “TB” for transmission-blocking, “MB” for mortality-blocking, and “RA” for recovery-augmenting. In the first three rows, the dashed gray lines denote the probability of the outcome for the nonadaptive case as a visual reference point, with one line for each scale of transmission modeled. In the last row, we do not plot any time to ER when there is no adaptation (“N”) or when ER does not occur. The columns indicate different host responses following infection, from 100% mortality (SI structure, *Left* column) to possible recovery (SIS structure, *Middle* column) to possible immunity (SIR structure, *Right* column) and point shape gives the scale of transmission. Squares indicate local “Density only” transmission, filled circles include both local density-dependent transmission and an implicitly modeled filled circles denote “Density w/reservoir”, which includes both local density-dependent transmission and an implicitly modeled reservoir (constant, additive probability of infection, regardless of number of infected individual present, allowing infection to re-emerge), and open circles denote the “Lasting disease shock” case (all nonadapted individuals are instantly and permanently infected; see details in *Methods* and *SI Appendix*). To make the adaptive pathways comparable, we model the adaptive allele as a 100% decrease (for transmission-blocking and mortality-blocking alleles) or 100% increase (for recovery-augmenting alleles) in the rate of the event, with dominance for heterozygotes at 50%. For the “Lasting disease shock case” we use these same rates for homozygote mutant-types as for the other transmission cases and set the heterozygote rates such that the probability of the respective event for heterozygotes is the mean between the probability of homozygous wild- and mutant-type individuals. The line range around each point gives the 95% quantiles of between-simulation differences (approximately 0.03 for the probability-based outcomes, *SI Appendix*, Fig. S1). (*C*) Parameter importance (permutation method) indicated by the global sensitivity analysis, computed via a random forest analysis: extinction (*Left* column, dark pink), ER (including both persistent and transient trajectories; *Middle* column, dark orange), and IL (*Right* column, light cream). The top six most important parameters are listed here (“res. transmission” is strength of transmission from the reservoir; “disease gens.” is number of disease generations between host reproduction events; trait SD is the imperfect inheritance around traits). The full importance ranking is in *SI Appendix*, Fig. S3.

The three main outcomes of our model, 1) extinction, 2) ER, and 3) IL without ER, show distinct population trajectories (Fig. 2*A*). We differentiate between ER and IL by identifying when the population recovers due to an increase in the adaptive allele (ER) or due to loss of disease (adaptive allele is rare and disease is extirpated; IL). Within the ER category, we identify both “Persistent” and “Temporary” trajectories. The “Persistent” ER case is more consistent with classic diagnostic features of ER, where the adaptive allele sweeps and population recovers to its historical carrying capacity (12), Fig. 2*A*, dark orange lines, “Persistent ER”]. Alternatively, for the “Temporary” ER case, population adaptation to disease can facilitate the loss of disease. Then, after disease is lost and the population recovers, the population might lose the adaptive allele (Fig. 2*A*, dark red-orange lines, “Temporary ER”). Thus, while there is a peak in the adaptive allele that enables ER, in the long-term, the allele does not persist. Temporary ER is rare compared to persistent ER in the parameter space we explore in the Main Text; we thus present the overall likelihood of ER in our Main Text figures (“All ER” category).

### Adaptation and Disease Characteristics Jointly Shape Population Trajectory.

Extinction risk is generally higher in the absence of adaptation (Fig. 2*B*, “N” adaptive pathway) and for infectious diseases that exhibit both density-dependent transmission and external transmission from a reservoir, compared to diseases with local density-dependent transmission only (Fig. 2*B*, point shape). Extinction is least likely when hosts can recover with immunity from infection, compared to when host response potential is limited to mortality or recovery (Fig. 2*B*, *Right* column compared to *Left* and *Middle* columns, respectively). Our GSA similarly highlights host response potential and adaptive pathway as two high-ranking factors for classifying outcomes across extinction, ER, IL, and no risk (Fig. 2*C*).

**Fig. 3.**
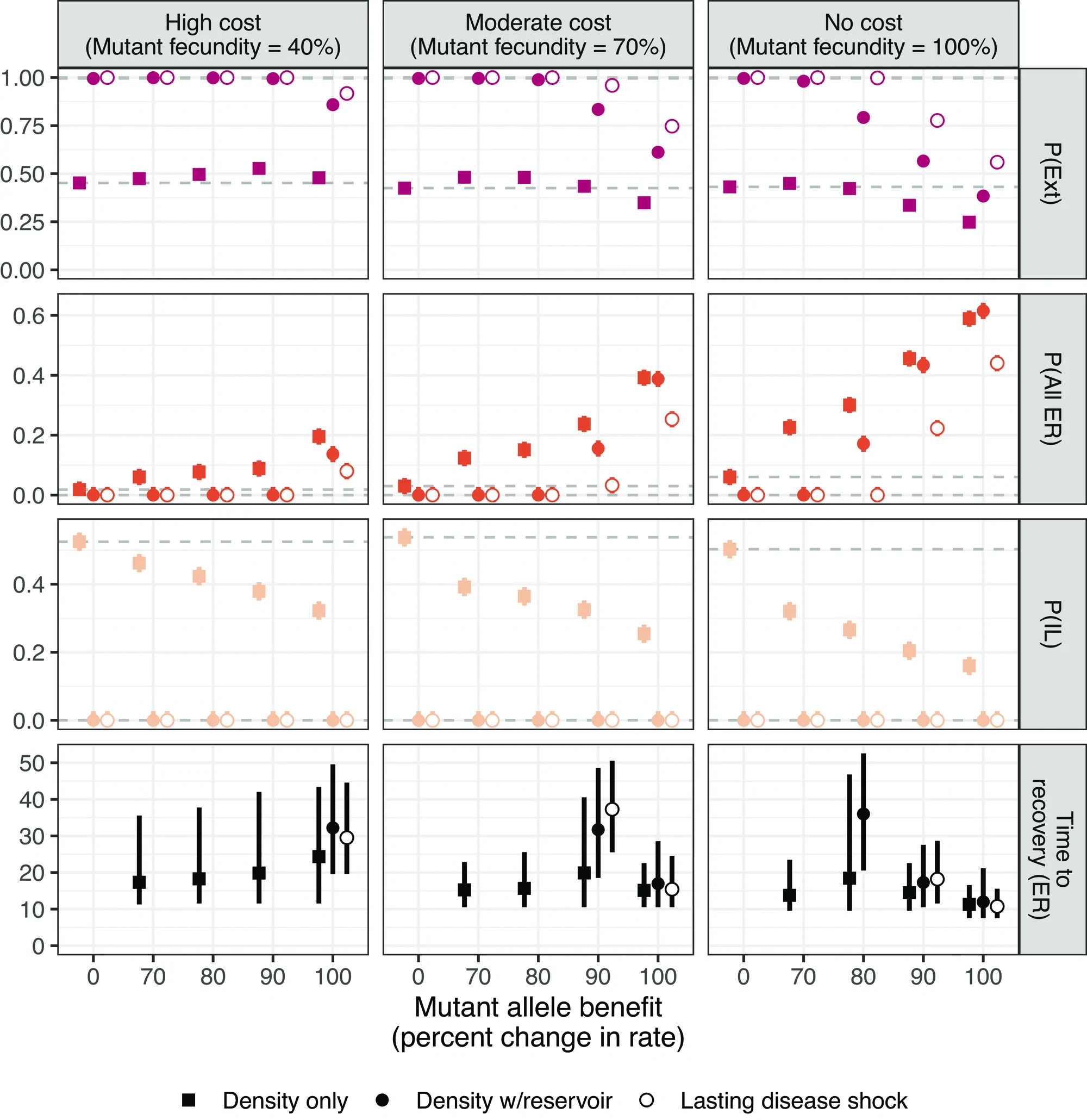
Probability of population outcomes (point color) under different adaptive pathways: extinction (*Top* row, dark pink), ER (including both persistent and transient trajectories; second row, dark orange), IL (third row, light cream), and time to recovery via ER (*Bottom* row, black). The x-axis denotes different degrees of the allele’s benefit. The benefit refers to the percent change (here, for mortality-blocking alleles, a decrease) in the rate of mortality. In the first three rows, the dashed gray lines denote the probability of the outcome for the nonadaptive case as a visual reference point, with one line for each scale of transmission modeled. In the last row, we do not plot any time to ER when there is no adaptation (“0” benefit) or when ER does not occur. The columns indicate different allele costs, measured as the proportionate fecundity a homozygous mutant individual has compared to a homozygous wild-type individual, from 40% fecundity (*Left* column) to 70% fecundity (*Middle* column) to 100% fecundity (no cost; *Right* column) and point shape denotes the scale of transmission. Squares indicate local filled circles denote “Density w/reservoir”, which includes both local density-dependent transmission and an implicitly modeled reservoir (constant, additive probability of infection, regardless of number of infected individual present, allowing infection to re-emerge), and open circles denote the “Lasting disease shock” case (all nonadapted individuals are instantly and permanently infected; see details in *Methods* and *SI Appendix*). These simulations assume hosts can recover without immunity following infection (SIS model structure). The line range around each point gives the 95% quantiles of between-simulation differences (approximately 0.03 for the probability-based outcomes, *SI Appendix*, Fig. S1).

The probability that a population will experience ER is generally greater when the adaptive allele confers tolerance, compared when the adaptive allele confers resistance (Fig. 2*B*, “MB” adaptive pathway versus “TB” adaptive pathway). Moreover, ER occurs more quickly as the cost of these alleles decreases (Fig. 3, *Right* column). When there is potential for disease re-emergence (“Density w/reservoir” case, Fig. 2*B*, filled circles), the likelihood of ER increases with increasingly optimistic host response potential for a given adaptive pathway, moving from 100% mortality to possible recovery with immunity (note for the “Density only” case with possible recovery with immunity “SIR” structure, the mechanism of population recovery primarily shifts to IL). The time to recovery via ER is generally comparable across adaptive pathways and host response potentials, controlling for allele benefit, allele cost, and population size (Fig. 2*B*, *Bottom* row).

IL occurs when there is local density-dependent transmission only (Fig. 2*B*, filled squares, third row). In this case, as the disease spreads, population declines decrease density-dependent transmission, which increases the probability that disease is stochastically extirpated. In our model, in the absence of an external reservoir, disease is only introduced once, so the loss of disease removes the ecological stressor and allows the population to recover. Our full GSA results affirm that a lack of or exceedingly rare contact with a reservoir, as well as structural factors like compartment structure and number of disease generations per host generation, influence the likelihood of IL (Fig. 2*C* and *SI Appendix*, Figs. S3 and S5).

### Scale of Transmission Shapes Trade-Offs Between Extinction, ER, and IL.

In line with the ER literature (11, 37), decreasing the cost or increasing the benefit of an adaptive allele increases the probability and speed of rescue (Fig. 3, second and fourth rows). However, the scale of transmission determines if the allelic properties that increase the likelihood of ER also reduce extinction risk. For closed populations experiencing local density-dependent transmission only, when the adaptive allele is costly (Fig. 3, *Left* and *Middle* column, filled squares), the likelihood of extinction can increase slightly even as the adaptive allele becomes more beneficial. This is because, as the adaptive allele begins to spread, IL becomes less likely and the population size is repressed for longer periods. In contrast, when disease can re-emerge from a reservoir (i.e., populations for which persistent IL is not possible), increasingly likely ER corresponds to decreasing extinction risk. For ER to occur, the allele must confer a strong benefit and have at most a moderate cost (Fig. 3). As compared to a counterfactual “Lasting disease shock” case (akin to a sudden, abiotic stress, Fig. 3, open circles), ER is more likely in the case with dynamical infectious disease transmission, given a fixed allele cost- and benefit. This suggests that stressors like infectious disease, which are endogenous to the system and affect a subset of the population at any one time, are more likely to facilitate ER versus an abiotic stressor that affects all individuals simultaneously and permanently.

### Large Populations Have the Highest Rescue Probability.

**Fig. 4.**
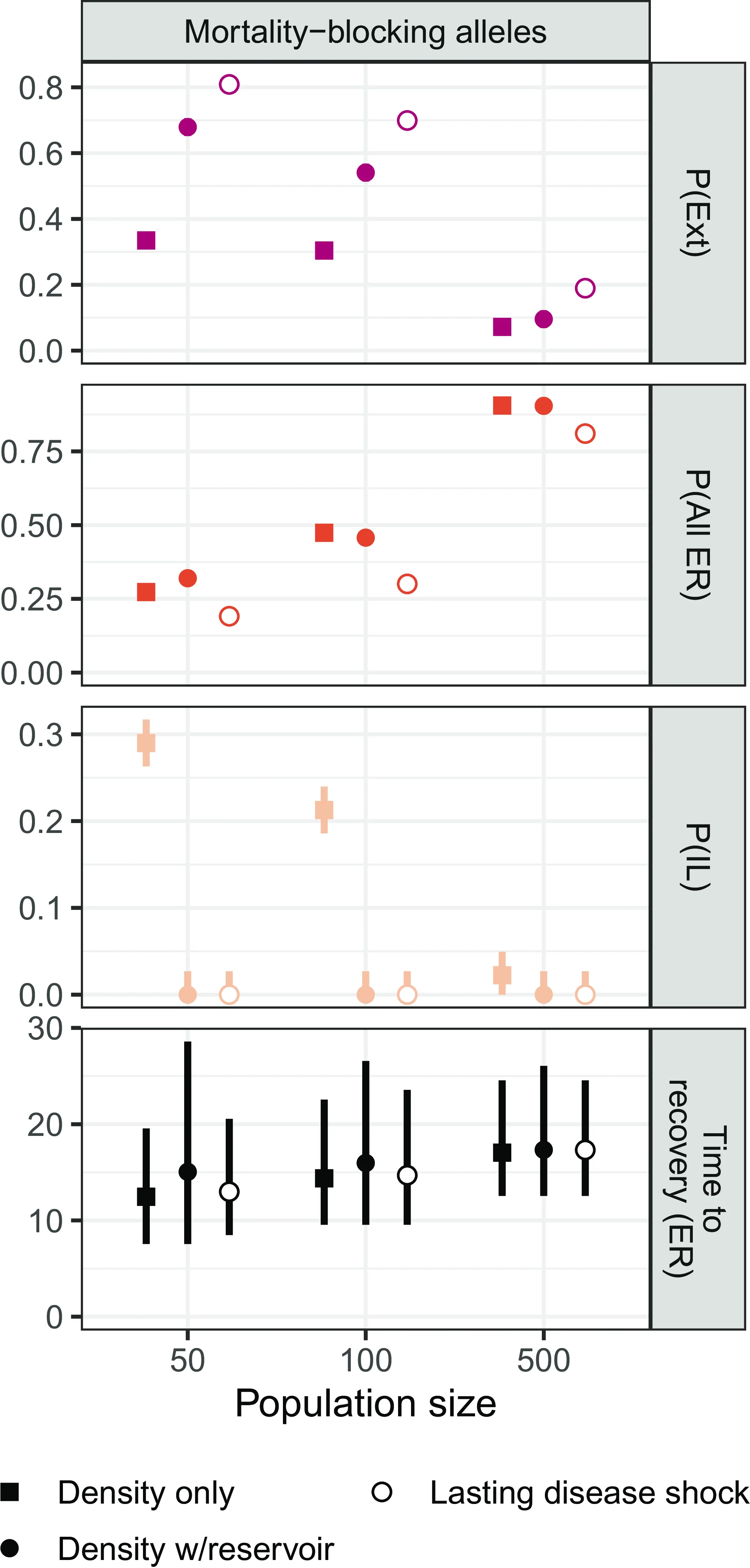
Probability of population outcomes (point color) under different adaptive pathways: extinction (*Top* row, dark pink), ER (including both persistent and transient trajectories; second row, dark orange), IL (third row, light cream), and time to recovery via ER (*Bottom* row, black). The x-axis denotes different maximum population sizes (i.e., carrying capacities). The point shape denotes the scale of transmission. Squares indicate local filled circles denote “Density w/reservoir”, which includes both local density-dependent transmission and an implicitly modeled reservoir (constant, additive probability of infection, regardless of number of infected individual present, allowing infection to re-emerge), and open circles denote the “Lasting disease shock” case (all nonadapted individuals are instantly and permanently infected; see details in *Methods* and *SI Appendix*). These simulations assume hosts can recovery without immunity following infection (SIS model structure) and evolve mortality-blocking alleles. The line range around each point gives the 95% quantiles of between-simulation differences (approximately 0.03 for the probability-based outcomes, *SI Appendix*, Fig. S1).

We find that larger populations exhibit lower extinction risk and higher probabilities of ER (Fig. 4, first and second rows, respectively) even though they tend to facilitate larger disease outbreaks (measured by higher average maximum disease prevalence). While stochasticity can be detrimental to small populations when it causes the loss of the adaptive allele, it can also benefit a declining population by inducing IL. As maximum population size increases, the likelihood of IL decreases (Fig. 4, third row). Larger populations are more buffered from the detrimental consequences of drift and have more mutations, which help facilitate ER and population recovery despite the decreased likelihood of IL. However, larger populations can take longer on average to recover to their carrying capacity because the magnitude of the population decline (and subsequent recovery) is larger (Fig. 4, *Bottom* row), though this effect is relatively small.

## Discussion

We show that stressors modeled after dynamical, infectious disease transmission result in a higher likelihood of ER compared to abiotic stressors that permanently affect all individuals, conditioned on the same adaptive allele architecture, benefit and cost. This contrasts slightly with past work on temporally varying stressors that predominantly focuses on exogenous environmental stochasticity, which shows that stochasticity can increase extinction risk relative to deterministic, constant abiotic stress (45, 46). The difference between our model results and these past results likely arises because we model an endogenous stress with dynamical, infectious disease-like transmission rather than an exogenous, stochastic, abiotic stress. In the endogenous case, some individuals do not experience stress and can thus help maintain population growth. In the exogenous case, more extreme years dampen population growth and prevent ER. These differences, in conjunction with immune-related adaptations that predominantly result from single-locus or large-effect mutations [e.g., frogs adapting to *Bd* fungus; (18, 19)], could help explain why conservation-focused ER is more often documented in infectious disease systems than in response to other abiotic stressors (13).

Among the scenarios explored here, we find that ER occurs most often when adaptive alleles have a low cost and confer disease tolerance (mortality-blocking; Figs. 2*B* and 3). ER is also more common when transmission is maintained via a reservoir. In contrast, diseases that spread via local density-dependent transmission only tend to lead to IL and result in relatively low extinction risk compared to other transmission scenarios [Fig. 2*B*; (47)]. We also show the possibility of “evolutionary suicide” [*sensu* (48)] for closed populations that evolve high-cost mortality-blocking alleles in response to diseases that spread via local density-dependent transmission only. However, the parameter space for this phenomenon is likely quite slim and future research efforts could quantify the degree to which this occurs in wild populations. Our results complement case-specific modeling work that highlights both ER and IL as mechanisms for population recovery (38, 39, 42) and align with work showing that different adaptive pathways can lead to host-pathogen coexistence or pathogen extirpation (34). We show that the generality of pathogen-driven ER and extinction depend heavily on relative generation times of host and pathogen reproduction, the adaptive pathway, and the host response potential (Fig. 2 and *SI Appendix*, Fig. S5).

Our results largely align with observed examples of ER in field contexts. For example, some frog populations are recovering from *Bd* fungus, a disease in part transmitted by reservoir species, through the spread of mortality-blocking adaptive alleles that confer tolerance (19). Moreover, *Bd* fungus has persisted in recovered populations (5), matching patterns of host-pathogen coexistence observed in our model. Similarly, mortality-blocking alleles in bats have enabled population recovery (7). For lake stickleback adapting to macroparasites, studies demonstrate that some populations develop transmission-blocking alleles, which reduce infection prevalence, and spur repeated loss and gain of the adaptive allele (25). These findings align with the dynamics we note in the “Temporary ER” case.

Our comparison of dynamical disease transmission to a counterfactual “Lasting disease shock” case more similar to an abiotic stress reinforces a key theme in the ER literature: Reducing environmental stress increases the likelihood of ER (13), for sudden and lasting stress (11), gradually changing environments (37), and environments with exogenous stochasticity (45, 46). We expand on this theme by pointing out that another pathway to reducing stress might include protecting some, but not necessarily all, individuals from stress. This would help mitigate the effects of stress on the average population growth rate, while still allowing selection to occur. This is similar to work showing that habitat heterogeneity affects the likelihood of ER by maintaining some nonaffected patches (49, 50). Protecting a subset of the population has possible draw backs, such as slowed efficiency of selection, that could be explored further by future work to identify the specific contexts that amplify or minimize this risk (27). Stress that fluctuates in time can lead to rounds of selection for and against adaptive alleles (25), possibly lengthening the time to population recovery. In some cases, this can lead to “Temporary ER,” whereby the evolution of the adaptive allele facilitates extirpation of the disease, which then prompts selection against the adaptive allele. Depending on the timescale considered, this might occur once (e.g., when there is local density-dependent transmission only) or be part of a larger pattern that slows or makes more variable the time to recovery via ER. Time to recovery can be slow when alleles are not fully protective or are costly and when the disease is reintroduced via a reservoir (Fig. 3). This finding differs slightly from past work, e.g., ref. 34, because we model density-dependent transmission with some additional infection from an external source (constant probability of infection).

As with all models, we make a number of assumptions to focus on the simplest possible model that captures the dynamics essential to our core question. We discuss three major assumptions here. First, we model disease dynamics as ordered, discrete events and ignore population structure. Using a discrete time representation of disease (transmission, mortality, recovery events) artificially changes the impacts of disease on selection because events are ordered and occur once per disease generation. When transmission occurs after mortality, extinction risk is generally lowered (conditioned on the number of disease generations; *SI Appendix*, Fig. S5). Increasing numbers of disease generations per host generation results in a higher risk of extinction and, when ER occurs, facilitates more rapid ER, because mortality and selection are stronger per host generation (*SI Appendix*, Fig. S5). We implicitly model a reservoir that can reintroduce disease, rather than explicitly modeling the reservoir by modeling a larger metapopulation of the same species (40, 41), an environmental transmission route or a second species that is more tolerant to disease. Explicitly modeling the dynamics of each of these cases could allow for more complex changes in selection pressure and affect the likelihood of ER.

Second, we model a single gene and assume the adaptive allele has pleiotropic effects on robustness against disease and reproduction. With reference to genetic architecture, some systems support the role of immune-related supertypes in promoting robustness against disease [e.g., frogs, (18, 19); abalone, (21, 22)], while in other systems, robustness against disease is multilocus [e.g., bats, (7); Tasmanian devils, (14, 15)]. Theory indicates that, when adaptation is the emergent result of multiple small-effect alleles, ER is less likely, though this depends on the relative evenness of effect sizes among alleles (51). More flexible genetic architectures could also moderate the trajectories of ER that we observe. For example, if a costly large-effect allele rescues the population in the short term, compensatory Adaptation later could help to reduce the long-term cost of adaptation (52, 53).

Third, given our focus on host ER, we do not consider coevolution of the pathogen. Considering pathogen coevolution could result in red queen dynamics (54, 55), whereby the host undergoes ER, and in response, the pathogen itself undergoes ER (discussed by ref. 36). In this scenario, we would expect to observe a lower likelihood of IL because pathogens could evolve to avoid their own extirpation. Among ER scenarios, when ER results in host-pathogen coexistence, selection on the parasite would likely be minimal. However, coevolution and ER of the parasite could still be relevant if disease prevalence decreases sharply.

Despite these limitations, our results create a framework to help managers identify possible conservation actions that lead to population recovery in the context of host-pathogen systems. We demonstrate that, because of dynamical disease transmission, ER in response to infectious disease is more likely compared to cases where all wild-type individuals are suddenly and permanently affected (“Lasting disease shock” case). This, coupled with the decreasing risk of extinction as host response potential moves from mortality to immunity (Fig. 2), indicates that efforts to either reduce extinction risk or slow the spread of infectious disease could help increase the likelihood of population recovery via ER or IL (2); see refs. 56 and 57 for species-specific interventions and management plans aiming to increase ER, including predator removal to reduce host mortality, habitat protection, and treatment]. That said, some minimal infectious disease prevalence is necessary to facilitate selection and ER: Very rapid extirpation of disease when there are reservoirs present could prevent selection and leave the population vulnerable to disease-mediated declines across longer timescales (27). When ER is feasible or likely, monitoring the effects of management interventions and iteratively updating management strategies in response to changes in ecoevolutionary dynamics can help managers weigh these risks (58). Managers could promote ER by introducing adaptive alleles through gene flow (59) and reducing extinction risk via habitat conservation or by reducing additional stressors (2); Figs. 3 and 4].

## Supplementary Material

Appendix 01 (PDF)

## Data Availability

Model code is available on GitHub under the repository lauriebalstad/hostpath_ER (60).
