# Supplementary material for: Drivers of the likelihood of evolutionary rescue in response to novel infectious diseases
Source: Proc Natl Acad Sci U S A. 2026 Oct 15;123(42):e2614169123. doi: 10.1073/pnas.2614169123 (PMC13684229; doi:10.1073/pnas.2614169123)
Supplement: Supplementary file 1 [file pnas.2614169123.sapp.pdf]

Supporting Information for

Drivers of the likelihood of evolutionary rescue in response to novel infectious diseases

Laurinne J Balstad, Marissa L Baskett

Corresponding Author: Laurinne J Balstad.
E-mail: laurinne.balstad@ubc.ca

This PDF file includes:

Supporting text
Figs. S1 to S5
Table S1
SI References

Supporting Information Text

Detailed methods

Initialization and disease-free dynamics. We initialize the simulation with a population of individuals (P_0), equal in size to the average environmental carrying capacity (K). All individuals are susceptible (S) to disease (i.e., there are no infected individuals) and there is some initial frequency of the M allele (Q_0) within the population. Because there is no disease during this phase, individuals experience only natural mortality. An individual's natural mortality phenotype ($\mu_{j_{G,T=S}}$) is drawn from a zero-truncated normal distribution centered around the genotype-specific natural mortality rate $\mu_{G,T=S}$, with environmental variance $\sigma^2_{\mu,G,T=S}$, i.e., $\mu_{j_{G,T=S}} \sim \mathcal{N}(\mu_{G,T=S}, \sigma^2_{\mu,G,T=S})$. The probability of survival for each individual is then

$$p_{j_{G,T=S},m} = \mathrm{e}^{-\mu_{j_{G,T=S}}}, \qquad \text{[S1]}$$

where survival is the result of a weighted coin flip, Binomial($p_{j_{G,T=S},m}$).

Next, individuals reproduce following density-dependent reproduction with a stochastic carrying capacity (K_t), drawn from a normal distribution centered around the average carrying capacity K with some variation σ^2_K, such that $K_t \sim \mathcal{N}(K, \sigma^2_K)$. To simulate reproduction, each individual produces a stochastic number of gametes $2 * \lambda_{j_{G,T},t}$ given diploidy, informed by their genotype-specific reproduction output, λ_G with some individual stochasticity across time $\sigma^2_{\lambda,G}$ following a Poisson distribution, i.e., $\lambda_{j_{G,T},t} \sim$ Poisson($2 * \lambda_G$). Reproductive output varies by genotype given a fitness cost C to being robust to disease that decreases reproductive output, i.e., the genotype-specific mean reproductive output is $\lambda_{MM} = C\lambda_{WW}$ ($C = 1$ indicates no cost). Homozygous individuals produce gametes of their allele type. Heterozygous individuals produce half their gametes as M type and half their gametes as W type; in the case where heterozygous individuals produce an odd number of gametes, a fair coin flip determines the allele of the final gamete. During reproduction, each individual gamete mutates to the opposite type with mutation probability Binomial(ν). Finally, gametes are paired together to create offspring, O_t and those offspring compete for space given the number of parents (P_t) and the stochastic carrying capacity (K_t): the total number of surviving offspring is $\min(O_t, K_t - P_t)$. Note that if no individuals die within a disease generation, it is possible that $P_t > K_t$, but in this case, no offspring will survive. We allow a burn-in period (t_q/d, with d being the number of disease generations per host reproductive events) for the population to reach a quasi-equilibrium around the carrying capacity and reach selection-mutation balance in the absence of disease.

Modeling disease dynamics. We introduce disease by changing change $\max(1, I_0 P_t)$ randomly selected individual(s) from susceptible to infected, where I_0 is the initial proportion of infected individuals (for the "Lasting disease shock" case counterfactual, we instantly convert all individuals to infected, though see specific mathematical details below for the transmission-blocking allele case). During this phase, we simulate both disease dynamics and density dependent reproduction with mutation. Disease dynamics always include transmission and mortality, and, in SIS and SIR cases where hosts can recover, recovery. For our base case, we simulate mortality first (natural and additive disease mortality), followed by transmission, ending with recovery (when applicable), repeating the disease cycle three times (d, disease generations), and then followed by host reproduction; we also explore sensitivity to the ordering of disease events and number of disease generations (see Section). At the end of each disease generation, we track the number of individuals ($P_t = \sum j_{G,T}$), the infection prevalence ($V_t = \sum j_{G,T=I}/P_t$), and the frequency of the M allele ($Q_t = (2 * \sum j_{G=MM,T} + \sum j_{G=WM,T})/(2 * P_t)$). For all parameters that depend on the disease robustness genotype G, we randomly draw offspring phenotypic values around the genotype to model imperfect inheritance and random environmental variation; the phenotype for all disease-related parameters is fixed for an individual's lifetime, while reproductive output varies each time step and is dependent on genotype to capture the cost of disease robustness (Main Text Figure 1).

Disease transmission describes susceptible individuals becoming infected and can occur through density-dependent transmission ($B = D$), contact with an external source of disease, e.g., a reservoir (implicitly modeled as a constant probability of infection; $B = V$), or a combination of both. An individual's transmission rate phenotype for each transmission type ($\beta_{B,j_{G,T=S}}$) is drawn from a zero-truncated normal distribution centered around the genotype-specific natural transmission rate $\beta_{B,G,T}$, with environmental variance $\sigma^2_{\beta,B,G}$. For density-dependent transmission ($B = D$), this rate is scaled by the number of infected individuals in the population ($I_t = \sum_G j_{G,T=I}$). The total probability of transmission ($p_{j_{G,T=S},f}$), accounting for both density-dependent transmission and environmentally-derived infection, for each individual j is then:

$$p_{j_{G,T=S},f} = 1 - \mathrm{e}^{-(\beta_{V,j_{G,T=S}} + \beta_{D,j_{G,T=S}} I_t)}, \qquad \text{[S2]}$$

as derived by (1). For susceptible individuals, transmission to become an infected individual is the result of a weighted coin flip, Binomial($p_{j_{G,T=S},f}$). To model the "Lasting disease shock" case, we assume that the rate of transmission for both routes is infinitely large, resulting in the probability of infection being 1 for homozygous wild-type individuals.

Disease mortality is additive to natural mortality for infected individuals. An individual's additive disease mortality phenotype ($\mu_{j_{G,T=I}}$) is drawn from a zero-truncated normal distribution with genotype-specific disease mortality rate $\mu_{G,T=I}$ with variance $\sigma^2_{\mu,G,T=I}$. The probability of survival for each infected individual is then

$$p_{j_{G,T=I},m} = \mathrm{e}^{-(\mu_{j_{G,T=S}} + \mu_{j_{G,T=I}})} \qquad \text{[S3]}$$

where survival is the result of a weighted coin flip, Binomial($p_{j_{G,T=I},m}$). Susceptible and immune individuals only experience natural mortality, their survival is the result of a weighted coin flip Binomial($p_{j_{G,T=S},m}$) where $p_{j_{G,T=S},m}$ is as in Equation S1. To model the "Lasting disease shock" case, the probability of mortality is given by these same genotype-specific rates.

Disease recovery only applies for SIS and SIR model structures and describes an infected individual becoming susceptible or immune, respectively. An individual's recovery phenotype ($\gamma_{j_{G,T=I}}$) is drawn from a zero-truncated normal distribution with genotype-specific disease recovery rate $\gamma_{G,T=I}$ with variance $\sigma^2_{\gamma,G,T=I}$. The probability of recovery for each infected individual recovering is then

$$p_{j_{G,T=I},c} = 1 - \mathrm{e}^{-\gamma_{j_{G,T=I}}}, \qquad \text{[S4]}$$

where recovery is the result of a weighted coin flip, Binomial($p_{j_{G,T=I},c}$). To model the "Lasting disease shock" case, we assume that the rate of recovery is 0, resulting in the probability of recovery being 0 for homozygous wild-type individuals.

We consider three types of disease robustness adaptations, in addition to a case with no evolution. When alleles are transmission-blocking, they decrease an individual's probability of moving from susceptible to infected, $\beta_{B,MM,S} \leq \beta_{B,MW,S} \leq \beta_{B,WW,S}$. When alleles are mortality-blocking, they decrease the probability an individual dies when infected, $\mu_{MM,I} \leq \mu_{MW,I} \leq \mu_{WW,I}$. Finally, when alleles are recovery-augmenting (only in SIS and SIR cases), they increase the rate individuals recover, $\gamma_{WW,I} \leq \gamma_{WM,I} \leq \gamma_{MM,I}$. The benefit of the adaptive alleles is the change in the rate of the event, denoted N (e.g., for for mortality-blocking alleles, $(1 - N) = \mu_{MM,I}/\mu_{WW,I}$ and $\mu_{MM,I} = (1 - N) * \mu_{WW,I}$; for recovery-augmenting alleles, the benefit increases the rate, $\gamma_{MM} = (1 + N) * \gamma_{WW}$). Heterozygote disease robustness depends on the degree of dominance $0 \leq D \leq 1$, with $D = 0$ for dominance of the W allele and $D = 1$ for dominance of the M allele.

For the "Lasting disease shock" case, we hold the homozygous mutant rate the same as for the disease-like transmission case being simulated. We then calculate the probability of each event for the heterozygote to be between the homozygous wild- and mutant-types, given dominance; we do this because the heterozygote rate itself cannot be set directly given that the homozygous wild-type rate is undefined (e.g., infinite for transmission). After transmission, mortality and recovery, reproduction happens following the same density-dependent and mutation process as described for the disease-free phase. We model t_f/d disease generations with disease.

Identifying evolutionary rescue. We categorize the simulation outcomes based on 1) whether the population has decreased relative to the carrying capacity, 2) whether the population has gone extinct or, after decreasing to its minimum, has recovered to near the carrying capacity, 3) whether the M allele has increased in frequency at some point during the simulation and 4) whether the disease persists or has been extirpated (Main Text Figure 1C). We classify trajectories as ER when a population 1) declines below the *a priori* selected threshold, $P_\theta = 0.5K$, for at least three time steps and stays below the threshold for 80% of the time steps between the first and last occurrence of declining below P_θ, 2) recovers to at least $0.95K$ after reaching the population minimum and 3) when the frequency of the M allele increases above some *a priori* selected threshold, $Q_\theta = 0.45$. We subclassify ER as either "Persistent" or "Temporary" based on the final disease prevalence and the final M allele frequency. To identify the time to recovery via ER, we use the host generation for which the population first reaches 95% of the carrying capacity (K) after the population minimum. We classify trajectories as infection loss (IL) when a population 1) declines below P_θ for at least three time steps and stays below the threshold for 80% of the time steps between the first and last occurrence of declining below P_θ, 2) recovers to at least $0.95K$ after reaching the population minimum, 3) when the frequency of the M allele remains below Q_θ and 4) when disease is extirpated ($I_{t=t_f} = 0$). In some simulations, populations do not experience extinction risk, either because the disease does not establish (due to stochasticity) or because the infection is not detrimental at the population scale (due to low transmission or virulence). These two types of trajectories are combined into a "no risk" (NR) category reflecting that the introduction of disease did not lead to population decline. In other simulations, populations experience long transience (i.e., do not recover or go extinct during the model simulation period, $t_q + t_f$). We classify these trajectories as "inconclusive".

We explored three alternative cut-offs each for P_θ and Q_θ, with strict ($P_\theta = 0.2K_t$ and $Q_\theta = 0.75$) , moderate ($P_\theta = 0.5K_t$ and $Q_\theta = 0.45$) and more permissive ($P_\theta = 0.8K_t$ and $Q_\theta = 0.15$) definitions of ER. Preliminary model explorations showed results were qualitatively similar across all three quantitative definitions of ER; in the Main text, we use the moderate thresholds to determine if the population shows an ER trajectory. In addition, we record the maximum and final disease prevalence (V_{max}, V_{t_f}), the maximum and final M allele frequency (Q_{max}, Q_{t_f}) and the time to population recovery ($t_{0.95K}$), if applicable.

Simulation experiments. We consider alternative combinations of disease ecology structures and individual adaptations across a single set of parameters (Table S1). In this "base case", we assume that the adaptive allele provides complete adaptation (e.g., for a transmission-blocking allele, $\beta_{B,MM,T=S} = 0$) with a cost $C = 0.7$ and set dominance at $D = 0.5$, such that the risk of extinction is generally high in the absence of evolution and ER occurs often to be relevant (Main Text Figure 2). Next, we simulate the effects of a mortality-blocking allele, given various cost-benefit trade-offs and population sizes. We consider cases where adaptive alleles provide only partial benefits and/or are costly for the case when host can recover from infection (SIS compartment structure), across different scales of transmission. We explore sensitivity to different average carrying capacities for the case when host can recover from infection (SIS compartment structure), across different scales of transmission. For each experiment, we simulate each parameter combination 2500 times to calculate the probability of different population outcomes (e.g., ER, extinction, IL; Figure S1) and provide a distribution of population dynamics (e.g., V_{t_f}, Q_{t_f}) among cases with ER at the end of the simulation.

Finally, we quantify how the individual parameters affect the outcomes through a global sensitivity analysis (GSA; (2)) across disease ecology structures and parameter values. In the GSA, we allow the prototrophic allele to provide disease robustness across one, two or all three adaptive pathways simultaneously. We consider 2000 unique combinations of parameters and scenarios (e.g., compartment structures), drawn using Latin Hypercube Sampling with the bounds described in Table S1. We simulate each unique combination 2500 times and then summarize each combination by the probability of extinction and rescue. We use a multi-variate random forest analysis ((3); *randomForestSRC* package, (4)) with 1000 trees to identify parameter importance for the probability distribution of various population outcomes, including extinction, ER, IL, no risk (i.e., the population did not decline) and a catch-all "inconclusive" category for unclassified simulations. We choose input parameters such that they are uncorrelated, e.g., instead of regressing the outcomes with $\beta_{V,MM,S}$, $\beta_{V,WM,S}$ and $\beta_{V,WW,S}$, we regress with the summarized $\beta_{V,WW,S}$, adaptive pathway and allele benefit N. We use the permutation method to calculate importance. We completed all simulations and calculations in R Version 4.4.3 (5).

Model robustness

We provide various additional analyses here to demonstrate robustness of model results. First we demonstrate convergence of simulations to confirm we used a large enough number of replicates to capture the distribution of outcomes (Figure S1).

Second, we show the distribution of outcomes from our GSA simulations (Figure S2), the full importance ranking of parameters from the multi-variate random forest analysis (Figure S3) and comparison between full simulations and random forest predictions for the parameters in Main Text (Figure S4 compared to Main Text Figure 2B). Our GSA showed that the number of disease generations and the ordering of discrete transmission, mortality and recovery events were both influential parameters on the probability of extinction and ER. Generally, increasing the number of disease cycles between host reproductive events increases the likelihood of extinction (Figure S5) and decreases the likelihood of IL (Figure S5). Extirpation is more likely to occur and IL is more less likely to occur when transmission is the first disease dynamic event, compared to transmission occurring after mortality or recovery. The number of disease cycles has minimal effect on the probability of ER; and likelihood of ER is generally higher when transmission occurs first (Figure S5), because pathogen extirpation (IL) is less likely, allowing the selective force to persist.

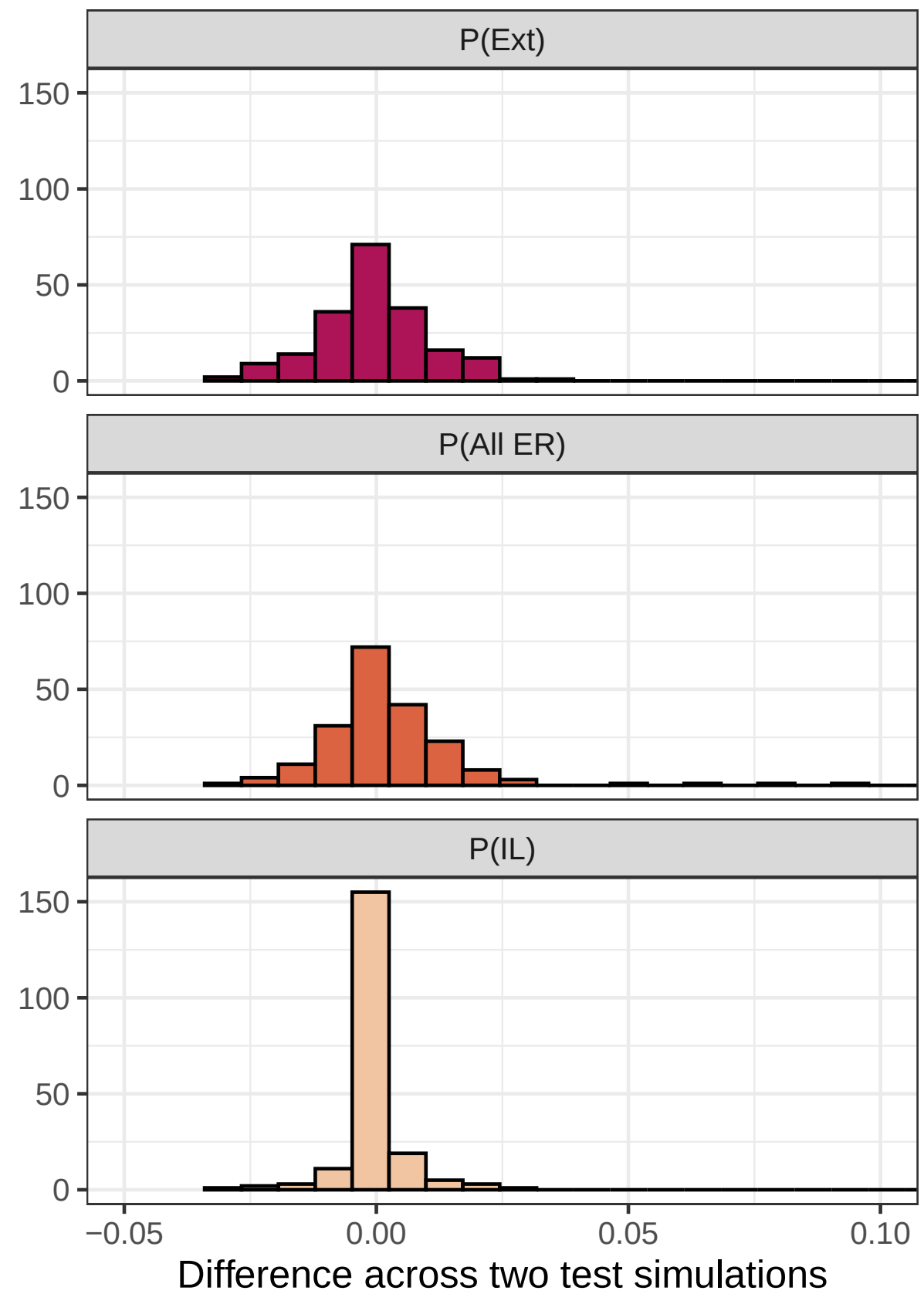

Fig. S1. Model convergence across 2500 simulations. Comparing point estimates from two sets of 2500 simulations for the parameters used across the Main text and supplement figures, excluding the simulations from the GSA. The 95% quartiles are -0.024-0.022, -0.019-0.027 and -0.014-0.011 for P(Ext) (top row), P(all ER) (middle row) and P(IL) (bottom row), respectively.

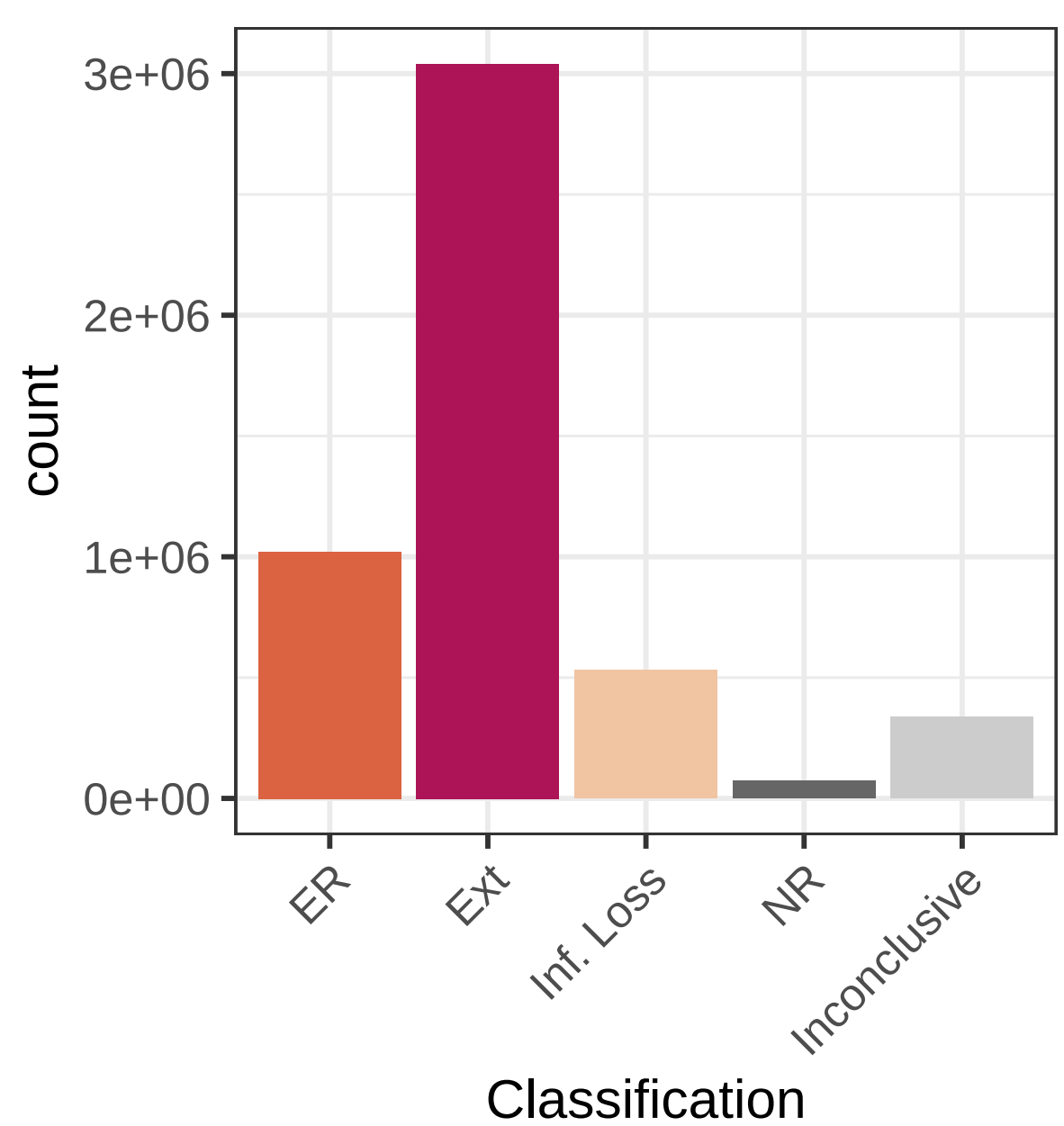

Fig. S2. The count of simulation outcomes (2000 parameter draws simulated 2500 times each) across the five outcomes of interest (L-R): the total probably of ER, extinction, IL, no response, and inconclusive.

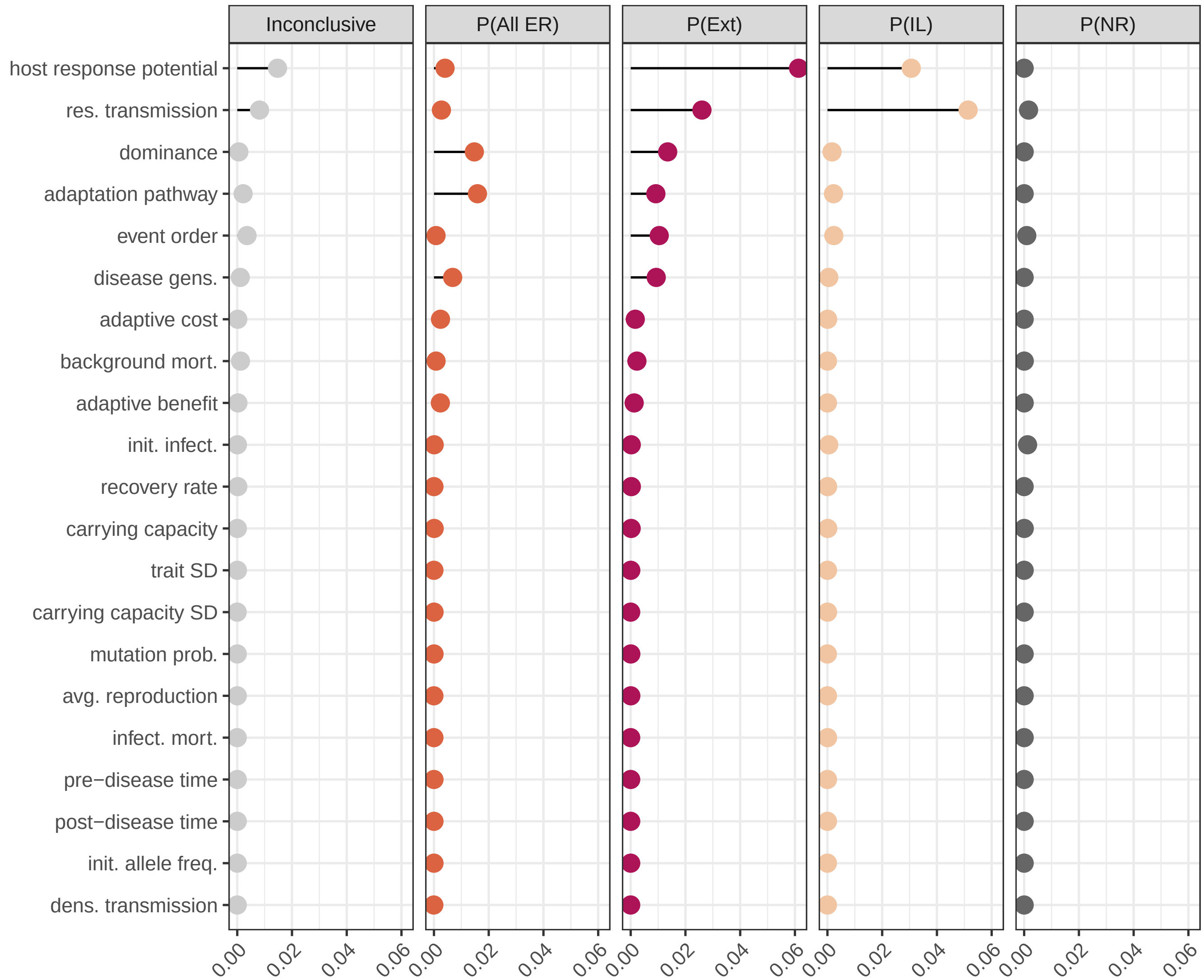

Fig. S3. Full importance estimates from the GSA across each outcome of interest. The point color indicates the event predicted (L-R): inconclusive, the total probably of ER, extinction, IL, and no response.

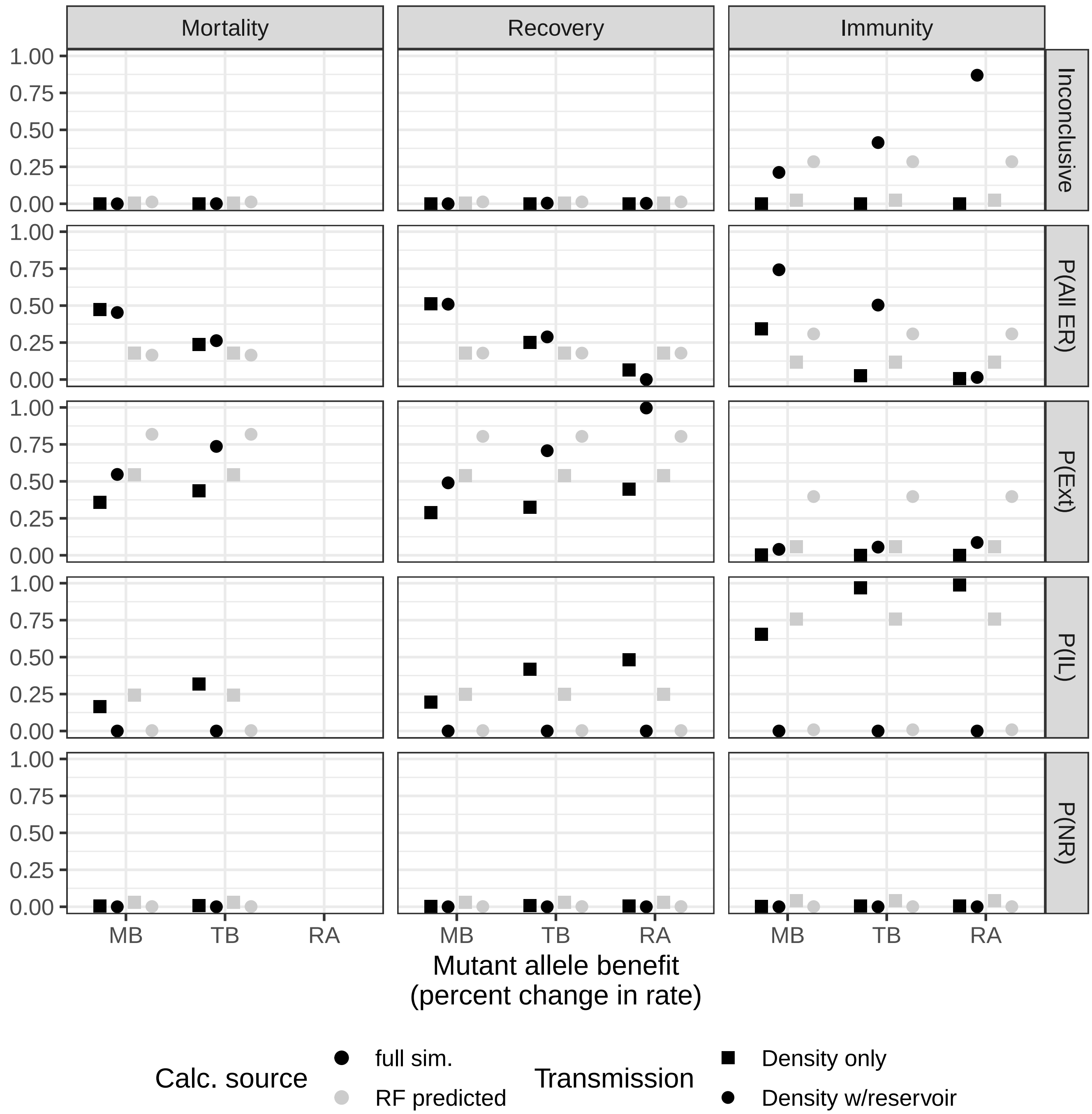

Fig. S4. A comparison of the Main Text Figure 2B outcomes with the predicted values from the random forest analysis. Probability of population outcomes under different adaptive pathways: inconclusive (top row), extinction (second row), ER (including both persistent and transient trajectories; third row), IL (fourth row), and no response (bottom row). The x-axis denotes the types of adaptive pathways, "TB" for transmission-blocking, "MB" for mortality-blocking, and "RA" for recovery-augmenting. Because we do not regress the random forest on the "none" adaptive pathway, we have excluded that pathway for this figure. The columns indicate different host responses following, from 100% mortality (SI structure; left column), to possible recovery (SIS structure; middle column), to possible immunity (SIR structure; right column) and point shape gives the scale of transmission. Squares indicate local "Density only" transmission, filled circles include both local density-dependent transmission and an implicitly modeled "Density w/reservoir" (constant, additive probability of infection, regardless of number of infected individuals, allowing infection to re-emerge) and open circles denote the "Lasting disease shock" case (all non-adapted individuals are instantly and permanently infected; see details in Methods section and Supplementary Materials). To make the adaptive pathways comparable, we model the adaptive allele as a 100% decrease (for transmission-blocking and mortality-blocking alleles) or 100% increase (for recovery-augmenting alleles) in rate of the event, with dominance for heterozygote at 50%. The point color indicates if it was calculated from the simulations (light gray) or predicted using the random forest regression (black).

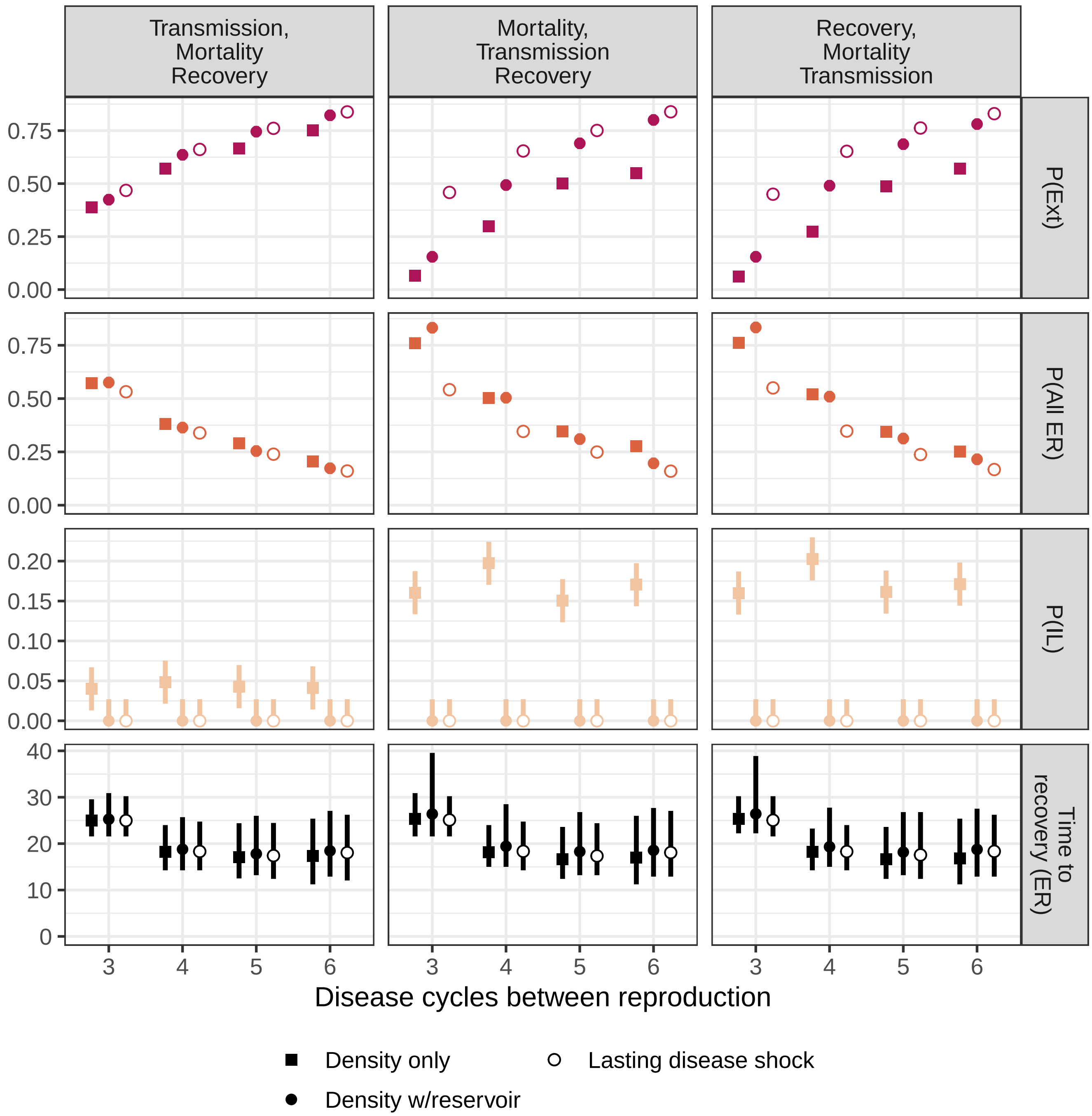

Fig. S5. Variation in discrete time structure. Probability of population outcomes (point color) under different adaptive pathways: extinction (top row; dark pink), ER (including both persistent and transient trajectories; second row; dark orange), IL (third row; light cream), and time to recovery via ER (bottom row; black). The x-axis denotes the number of disease generations between host generations. The columns indicate different orderings of disease events within a SIS compartment structure and point shape gives the scale of transmission. Squares indicate local "Density only" transmission, filled circles include both local density-dependent transmission and an implicitly modeled "Density w/reservoir" (constant, additive probability of infection, regardless of number of infected individuals, allowing infection to re-emerge) and open circles denote the "Lasting disease shock" case (all non-adapted individuals are instantly and permanently infected; see details in Methods section and Supplementary Materials). These simulations assume hosts can recovery without immunity following infection (SIS model structure) and evolve mortality-blocking alleles. The line range around each point gives the 95% quantiles of between-simulation differences (approximately 0.03 for the probability-based outcomes, Figure S1).

Table S1. Parameter definitions and values used in base case simulations. In the Main text, simulations always include density-dependent transmission, with the implicit reservoir transmission turned on or off. For $\beta_{D,WW,S}, \beta_{V,WW,S}, \gamma_{WW,I}$ the three parameters listed reflect, in order, the case where there is only density-dependent transmission, where there is both density-dependent transmission and an implicit reservoir, and where there is a "Lasting disease shock", respectively. Similarly, the Main text simulations consider only one adaptive pathway at a time, with the other adaptive pathways removed such that MM individuals have the same phenotype distributions as the wild-type, i.e., for adaptive pathways that are inactive, X_{MM} follows the same distribution as X_{WW}. Information about transmission pathways and adaptive pathways simulated are included in the figure captions. In the "GSA range" column, "N/A" indicates the variable was not varied and excluded from the GSA.

**State variable**	**Meaning**	**Possible values**		
P_t	Total population size at time t	$0 \leq P_t$		
O_t	Offspring population size at time t	$0 \leq O_t$		
**Individual attributes**	**Meaning**	**Possible values**		
G	Individual genotype	WW, WM, MM		
T	Individual infection status	S, I, R		
**Parameter**	**Meaning**	**"Base case" value**	**Units**	**GSA range**
–	Compartment structure simulated	SIS		SI, SIS, SIR
–	Adaptive pathway simulated (Transmission-blocking (TB); Mortality-blocking (MB); Recovery-augmenting (RA))	Varies		MB,TB,RA, MB+TB, MB+RA, TB+RA, MB+TB+RA
B	Transmission route simulated	–		D, V, D+V
t_q	Burn-in period	160	disease generations	N/A
t_f	Simulation period	160	disease generations	N/A
d	Disease generations between host reproductive events	3	disease generations	2-6
Q_0	Frequency of M allele at start of burn-in period	0.05		N/A
I_0	Proportion of infected individuals at start of simulation period	0.1		0.05-0.15
K	Mean carrying capacity	100	individuals	70-120
σ_K^2	Variation in carrying capacity	4		3-7
ν	Mutation probability	0.005		0.0005-0.01
$\mu_{G,S}$	Mean natural host mortality rate	0.05	time^{-1}	0.03-0.06
N	Benefit of adaptive allele	1	Change in rate	0.925-1
$\beta_{D,WW,S}$	Mean density-dependent transmission rate for genotype WW	1, 1, Inf	time^{-1}	0.8-1.2
$\beta_{D,MM,S}$	Mean density-dependent transmission rate for genotype MM	0	time^{-1}	$\beta_{D,WW,S} * (1-N)$
$\beta_{V,WW,S}$	Mean environmental transmission rate for genotype WW	0, 0.05, Inf	time^{-1}	0-0.1
$\beta_{V,MM,S}$	Mean environmental transmission rate for genotype MM	0	time^{-1}	$\beta_{V,WW,S} * (1-N)$
$\mu_{WW,I}$	Mean virulence rate for genotype WW, additive to $\mu_{WW,S}$	0.95	time^{-1}	0.8-1.2
$\mu_{MM,I}$	Mean virulence rate for genotype MM, additive to $\mu_{MM,S}$	0	time^{-1}	$\mu_{WW,I} * (1-N)$
$\gamma_{WW,I}$	Mean recovery rate for genotype WW	0.05, 0.05, 0	time^{-1}	0.05-0.2, 0 for SI
$\gamma_{MM,I}$	Mean recovery rate for genotype MM, note $\gamma_{WW,I} < \gamma_{MM,I}$	0.1	time^{-1}	$\gamma_{MM,I} * (1+N)$
C	Cost of adaptive allele	0.7		0.5-1
λ_{WW}	Mean number of offspring per WW parent	1		1-1.2
λ_{MM}	Mean number of offspring per MM parent	0.7		$\lambda_{WW} * C$
$\sigma_{X,G}^2$	Environmental variance around genotypic means for events $X = \mu, \beta, \gamma$	0.01		0.01-0.1
D	Dominance of adaptive allele	0.5		0-1

References

1. LJ Allen, P van den Driessche, The basic reproduction number in some discrete-time epidemic models. *J. difference equations applications* **14**, 1127–1147 (2008).
2. EB Harper, JC Stella, AK Fremier, Global sensitivity analysis for complex ecological models: a case study of riparian cottonwood population dynamics. *Ecol. Appl.* **21**, 1225–1240 (2011).
3. L Breiman, Random forests. *Mach. learning* **45**, 5–32 (2001).
4. H Ishwaran, UB Kogalur, EH Blackstone, MS Lauer, *Random survival forests*, (2008).
5. R Core Team, *R: A Language and Environment for Statistical Computing* (R Foundation for Statistical Computing, Vienna, Austria), (2025).
